# Polyol and sugar osmolytes can shorten protein hydrogen bonds to modulate function

**DOI:** 10.1038/s42003-020-01260-1

**Published:** 2020-09-23

**Authors:** Jingwen Li, Jingfei Chen, Liaoyuan An, Xiaoxiang Yuan, Lishan Yao

**Affiliations:** 1 Key Laboratory of Biofuels, Qingdao Institute of Bioenergy and Bioprocess Technology, Chinese Academy of Sciences, Qingdao, 266101 China; 2 Shandong Provincial Key Laboratory of Synthetic Biology, Qingdao Institute of Bioenergy and Bioprocess Technology, Chinese Academy of Sciences, Qingdao, 266101 China; 3 University of Chinese Academy of Sciences, Beijing, 100049 China

**Keywords:** Molecular biophysics, Computational biology and bioinformatics

## Abstract

Polyol and sugar osmolytes are commonly used in therapeutic protein formulations. How they may affect protein structure and function is an important question. In this work, through NMR measurements, we show that glycerol and sorbitol (polyols), as well as glucose (sugar), can shorten protein backbone hydrogen bonds. The hydrogen bond shortening is also captured by molecular dynamics simulations, which suggest a hydrogen bond competition mechanism. Specifically, osmolytes weaken hydrogen bonds between the protein and solvent to strengthen those within the protein. Although the hydrogen bond change is small, with the average experimental cross hydrogen bond ^3h^*J*_NC′_ coupling of two proteins GB3 and TTHA increased by ~ 0.01 Hz by the three osmolytes (160 g/L), its effect on protein function should not be overlooked. This is exemplified by the PDZ3−peptide binding where several intermolecular hydrogen bonds are formed and osmolytes shift the equilibrium towards the bound state.

## Abstract (web summary)

With NMR and MD simulations, Li, Chen et al. comprehensively probe the effects of osmolytes on hydrogen bond formation in proteins. They show that polyol and sugar osmolytes weaken hydrogen bonding between the protein and solvent while strengthening those within the protein.

## Introduction

Osmolytes are small molecules that are used by cells to counter the osmotic stress^1^. There are three different classes of osmolytes: polyols and sugars, amino acids and their derivatives, and methyl ammonium compounds^1^. Protecting osmolytes are able to increase protein stability and prevent protein aggregation^2–7^. These osmolytes stabilize the protein folding generally through the preferential exclusion mechanism^8–13^ where the exclusion of osmolytes on the protein surface increases the protein chemical potential. For the equilibrium between the native and denatured states, the increase in chemical potential is greater for the denatured state, which has a larger surface area. Hence, the free energy of denaturation is increased and the native state is stabilized^14–16^. The unfolded state is destabilized more because it has more solvent accessible surface areas than the folded state^4,17,18^. Furthermore, the protein backbone contributes more to the stabilization than the side chains due to its more unfavorable interaction with osmolytes^16^.

Osmolytes are commonly used in biopharmaceuticals because they are able to extend the shelf-life through protein stabilization^19^. More than 100 biological formulations were approved by the U.S. food and drug administration (FDA) between 1998 and 2017^19^. For example, polyols and sugars are frequently used in the formulation of vaccines^20,21^ and antibody drugs^5,22^. Trehalose is used in several commercial therapeutic formations, such as Herceptin^®^, Avastin^®^, Lucentis^®^, and Advate^®^^5^. The broad usage of osmolytes in therapeutic proteins raises a critical question: do they affect protein structure and function besides stabilizing the folded conformation? One would expect that the osmolytes effect on protein structure is small, because they are excluded from the protein surface. However, it has been reported recently that sucrose compresses the tertiary structure but has no effect on the secondary structure of the ribosomal protein S6^23^. It has also been shown that trehalose compresses bovine serum albumin and increases its α-helicity^24^. But the mechanism of the structure change has not been addressed. More studies are urgently needed on the topic.

Hydrogen bonds (H-bonds) are important for protein folding^25,26^. Backbone h-bonds between amide hydrogens and carbonyl oxygens are critical to maintain the α-helix and β-sheet secondary structures^27–29^. Furthermore, h-bonds can play an active role in enzyme catalysis by stabilizing the transition state, as demonstrated by several studies^30–32^. Depending on the role of h-bond, its small change can have an important effect on protein function. For example, in the catalysis by chymotrypsin which has a Ser–His–Asp triad active site, a small increase of the h-bond strength between His and Asp, e.g., 0.1 ppm chemical shift increase for the proton in the h-bond, can almost double the enzyme specificity (*k*_cat_/*K*_m_) against peptide substrates^33^. The significance of h-bonds prompts us to investigate whether osmolytes may affect the h-bond strength in proteins. Two proteins, GB3 (56 amino acids, the third IgG-binding domain from Streptococcal protein-G)^34^ and TTHA1718 (66 amino acids, a putative heavy metal-binding protein from *Thermus thermophilus*)^35^ were selected in this study. Glycerol, sorbitol, and glucose were selected as the representative polyol and sugar osmolytes. The backbone N−H…O=C h-bond strength was monitored by the cross-h-bond J-coupling constant, ^3h^*J*_NC′_, which can be measured by NMR with very high precision for small deuterated proteins^36–40^. The results show that the ^3h^*J*_NC′_ coupling constant is larger in the presence of osmolytes, suggesting that osmolytes strengthen the backbone N−H…O=C h-bond. Molecular dynamics simulations of GB3 in the absence or presence of osmolytes capture the experimental h-bond changing effect, based on which a h-bond competition mechanism is proposed. The change of the protein h-bond also affects the protein–ligand binding and the H/D exchange process. This is exemplified by the PDZ3 (the third PDZ domain of the neuronal signaling protein PSD-95/SAP90, residue 303−395) binding to a peptide where multiple intermolecular hydrogen bonds are formed^41^. In the presence of osmolytes, the binding becomes tighter and the H/D exchange rates are slower. Our study provides direct evidence that protein function can be modulated by osmolytes through perturbing h-bonds.

## Results

### Protein h-bond perturbation by osmolytes

**Fig. 1:**
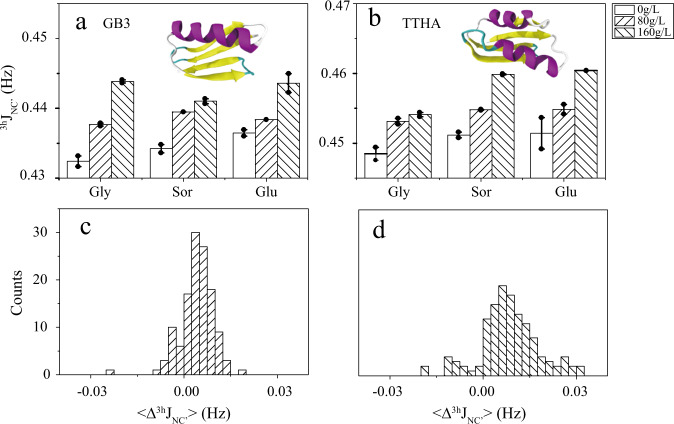
Effect of osmolytes on protein backbone h-bonds. The mean through h-bond *J*-coupling constant <^3h^*J*_NC′_> is the average ^3h^*J*_NC′_ (absolute value) in the absence or presence of osmolytes. <…> is the average over 27 and 15 h-bonds for two proteins GB3 (**a**) and TTHA (**b**), respectively. The positive <Δ^3h^*J*_NC′_> (<Δ^3h^*J*_NC′_> = <^3h^*J*_NC′_ (osmolyte_)_> – <^3h^*J*_NC′_ (buffer)>) indicates that the h-bonds are stronger on average in the presence of osmolytes. The histograms of site specific Δ^3h^*J*_NC′_ at 80 g/L (**c**), and 160 g/L (**d**) osmolyte concentrations suggest that most h-bonds are strengthened. The histogram was built based on the 42 h-bond changes for the two proteins by the three osmolytes at two concentrations. Abbreviations: Gly glycerol; Glu glucose; Sor sorbitol. The error bars on **a**, **b** are standard errors (S.E.) of the mean (*n* = 2 independent experiments).

Both GB3 and TTHA adopt a compact folding, with α-helix and β-strand secondary structure elements (Fig. 1). A total of 27 and 15 through backbone N−H…O=C h-bond ^3h^*J*_NC′_ coupling constants were measured for GB3 and TTHA, respectively using the pulse sequence developed by Cordier and Grzesiek^36^ (Fig. 1 and Supplementary Table 1). To improve the measurement accuracy, both proteins were deuterated and ^15^N/^13^C isotopically labeled and only well-resolved peaks in the 2D-H(N)CO spectra were picked and analyzed^36^. It is known that ^3h^*J*_NC′_ has a negative sign^42^, herein only its absolute value, as determined in the NMR experiments, is discussed. Addition of 80 g/L glycerol, sorbitol, and glucose to GB3 increases the average ^3h^*J*_NC′_ (<Δ^3h^*J*_NC′_> = <^3h^*J*_NC′_ (osmolyte)> − <^3h^*J*_NC′_ (buffer)>) by 0.005, 0.005, and 0.002 Hz, respectively. The positive <Δ^3h^*J*_NC′_> indicates that the average h-bond strength is enhanced. Raising the osmolytes concentration to 160 g/L increases the average ^3h^*J*_NC′_ by 0.011, 0.007, and 0.007 Hz. For TTHA, the same effect was observed. An early study by Cordier and Grzesiek shows that the ^3h^*J*_NC′_ J-coupling increases by 0.0017 Hz when the temperature decreases by 1 °C^38^. The ^3h^*J*_NC′_ changes measured for the two proteins with 160 g/L osmolytes suggest that the osmolytes have an effect equivalent to a temperature decrease of 3–6 °C. To see whether the average ^3h^*J*_NC′_ increase is caused by the change of just a few h-bonds or the majority of them, a histogram of the site-specific Δ^3h^*J*_NC′_ (two proteins together) is plotted. In the presence of 80 g/L osmolyte, 83% of h-bonds show a positive Δ^3h^*J*_NC′_. The percentage increases to 89% when the osmolyte concentration increases to 160 g/L. The two histograms clearly suggest that most h-bonds are stronger in the presence of the osmolytes. The Δ^3h^*J*_NC′_ change also indicates that the h-bond donor−acceptor (N−O) distance may have been perturbed. Based on an empirical equation proposed between the N−O distance and ^3h^*J*_NC′_, |^3h^*J*_NC′_| = 59,000 × exp(−4*R*_NO_)^38^ where *R*_NO_ is the N−O distance, it can be estimated that an increase of 0.01 Hz *J*-coupling constant (at ^3h^*J*_NC′_ of 0.4 Hz, close to the average of ^3h^*J*_NC′_ in the two proteins) corresponds to a decrease of the N−O h-bond distance by 0.006 Å. Although the structural change is extremely small, the NMR measurements are capable of capturing it.

**Fig. 2:**
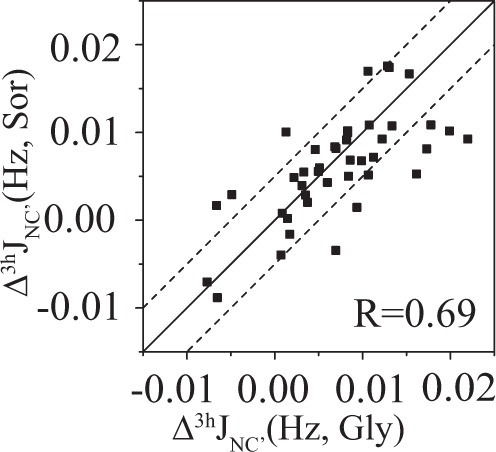
Correlation of site specific Δ^3h^*J*_NC′_ in different osmolytes. Δ^3h^*J*_NC′_ in sorbitol shows a moderate correlation with that in glycerol (Δ^3h^*J*_NC′_ is the average of the two Δ^3h^*J*_NC′_ values in 80 and 160 g/L osmolyte concentrations). The error of ^3h^*J*_NC′_ in different osmolytes is ~0.004−0.006 Hz. The two dashed lines are *y* = *x* ± 0.005 Hz, suggesting that the J-coupling deviation from the line *y* = *x* may be caused by the measurement error.

Site-specific Δ^3h^*J*_NC′_ values (averaged over 80 g/L and 160 g/L osmolyte concentrations) in different osmolytes are plotted against each other. Compared to the average Δ^3h^*J*_NC′_, the site-specific Δ^3h^*J*_NC′_ has a larger error, ~0.005 Hz, based on duplicate measurements. Even so, a moderate correlation is observed for Δ^3h^*J*_NC′_ in glycerol, sorbitol, and glucose (Fig. 2 and Table 1). This correlation suggests that glycerol, sorbitol, and glucose affect the protein h-bonds through a common mechanism. This is not fully surprising because glycerol, sorbitol, and glucose all have multiple hydroxyl groups. They may have a similar interaction property with protein surfaces. This will be discussed later.

**Table 1:** Pearson correlation coefficient (*R*_P_) of Δ^3h^*J*_NC′_ in different osmolytes.

	Gly	Sor	Glu
Gly	1	0.69	0.66
Sor		1	0.73
Glu			1

### Inter- and intra-molecular h-bond competition

**Fig. 3:**
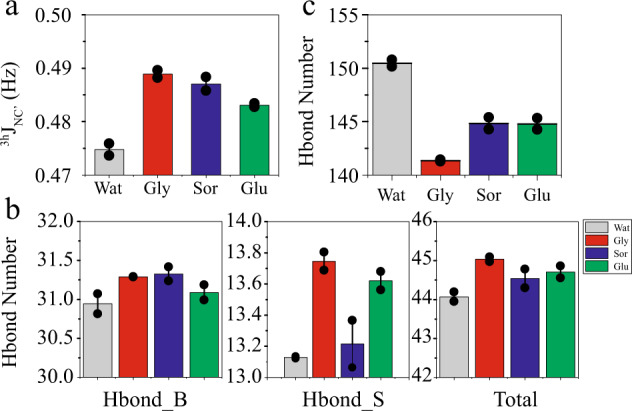
Effect of osmolytes on GB3 intramolecular and intermolecular (with solvent) h-bonds from MD simulations. **a** Average ^3h^*J*_NC′_ coupling constant change <Δ^3h^*J*_NC′_> caused by the three different osmolytes at the concentration of ~500 g/L. **b** Changes of the intramolecular h-bond number in pure water and in osmolytes. It shows that the number of backbone (Hbond_B) and side chain (Hbond_S) h-bond increases in all three osmolytes. **c** Number of protein–solvent intermolecular h-bond (protein–water + protein–osmolyte). It appears that osmolytes tend to weaken the protein–solvent intermolecular h-bond to enhance the protein intramolecular h-bond. The error bars are S.E. of the mean (*n* = 2 independent MD runs).

To further understand the h-bond stabilization mechanism by osmolytes, replica exchange molecular dynamics (REMD)^43^ simulations were performed for GB3 in pure water or in water with glycerol (500 g/L), sorbitol (530 g/L), or glucose (530 g/L) at the temperature of 300−360 K. The starting osmolyte concentration was set as the same (500 g/L). But the volume change of the rectangular box in the MD equilibration process causes the small osmolyte concentration difference which should have a very minor effect on the h-bond changes. More osmolytes (compared to the experimental concentration) were added in the simulations to magnify the structural perturbation effect. The trajectories at the temperature of 300 K were used for h-bond analyses. Consistent with the experimental results, the three osmolytes, glycerol, sorbitol, and glucose, increase the average ^3h^*J*_NC′_, by 0.014, 0.012, and 0.008 Hz, respectively, for the 27 experimentally measured backbone h-bonds in GB3 (Fig. 3a). The computational average Δ^3h^*J*_NC′_ of 0.011 Hz in the three osmolytes is ~40% larger than the corresponding experimental average at 160 g/L of osmolytes. Although quantitative data are difficult to predict, MD simulations do capture the average h-bond changing trend, which encourages us to dissect the h-bond perturbation mechanism further using the MD trajectories.

In the MD simulations, different types of h-bonds can be counted, which provide more insights into the stabilization mechanism. The number of protein backbone–backbone h-bonds is 30.9 in pure water, averaged over 100 ns MD trajectories. It increases by 0.35, 0.38, and 0.15 in glycerol, sorbitol, and glucose, respectively. Meanwhile, the protein side-chain h-bonds (including side-chain−side-chain and side-chain–backbone) increase in glycerol, sorbitol, and glucose by 0.62, 0.09, and 0.49 (Fig. 3b) from 13.1 in pure water. As a result, the total protein intramolecular h-bonds increase by 0.97, 0.47, and 0.64 in the presence of the three osmolytes, respectively. Furthermore, MD simulations provide information about protein solvent (water and osmolyte) h-bonds (Fig. 3c). In the presence of osmolytes, ~20−25% of protein solvent h-bonds are from the protein−osmolyte pair (Supplementary Table 2). The number of protein–solvent h-bonds drops considerably in glycerol, sorbitol, and glucose, compared to that in pure water (Fig. 3c). It appears that the weakening of the protein–solvent h-bonds stimulates the polar groups in the protein to form stronger intramolecular h-bonds. This h-bond competition mechanism is corroborative with an early computational study of osmolyte effects on a beta-hairpin folding and unfolding equilibrium^17^.

### Tighter protein–ligand binding by osmolytes

**Fig. 4:**
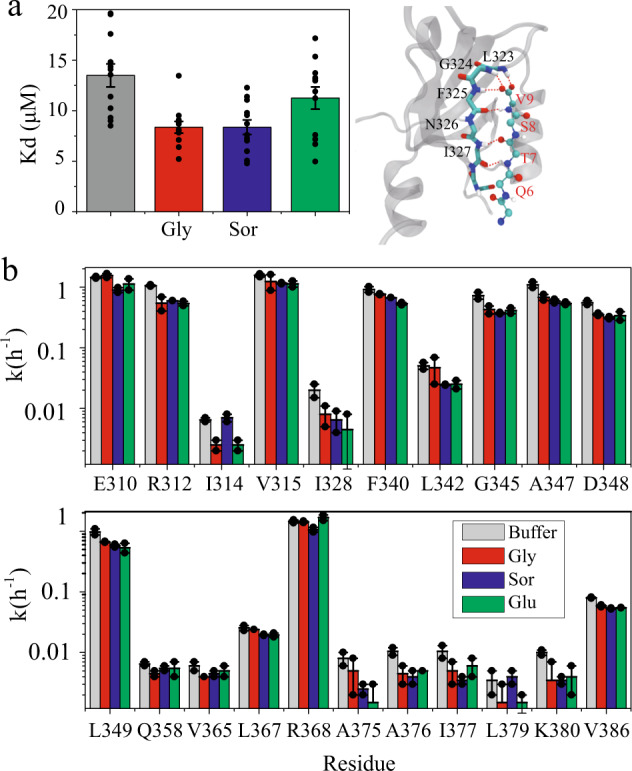
Osmolyte effect on protein ligand binding and protein h-bond. Osmolyte modulation of dissociation constant *K*_d_ (**a**) and H/D exchange rates *k* (**b**) of the PDZ3−CRIPT peptide complex. The right side of **a** shows that six h-bonds are formed between the backbones of PDZ3 (labeled in black) and CRIPT (labeled in red). The dissociation constant *K*_d_ was measured by a NMR titration experiment (Supplementary Fig. 1). The chemical shifts Δ*ω* (Δ*ω* = [(∆*ω*_H_)^2^ + (0.1*∆*ω*_N_) ^2^]^0.5^) of 12 backbone amides at different CRIPT concentrations were fitted to produce the binding constant for each site which was then averaged to yield the binding constant for the whole protein. The error bars are S.E. of the mean (*n* = 12 residue specific *K*_d_s). The H/D exchange rate (**b**) was measured by adding PDZ3−CRIPT powder (with the final concentration of 0.3 mM PDZ3 and 1 mM CRIPT) to a D_2_O buffer (50 mM phosphate at pH 7.0, in the absence or presence of 160 g/L osmolytes) and recording ^1^H-^15^N HSQC spectra consecutively (30 min per spectrum) for 12 h. In all, 21 residues show detectable H/D exchange rates which are generally smaller in the presence of osmolytes. The error bars are S.E. of the mean (*n* = 2 independent experiments).

The shortening of protein h-bonds in principle should facilitate the protein–ligand binding if extra h-bonds are formed between the two molecules in the complex form. This is tested by the binding measurement of PDZ3 (residue 303−395) and a CRIPT peptide (Ac-TKNYKQTSV-COOH)^41^. Six inter-molecular h-bonds are formed between the backbone of PDZ3 and the CRIPT peptide in the X-ray crystallography structure of the complex (Fig. 4)^41^. These h-bonds are essential for the PDZ3−CRIPT binding. The dissociation constant *K*_d_ was measured for the PDZ3−CRIPT complex using the NMR chemical shift titration experiment in the absence or presence of different osmolytes (Supplementary Fig. 1). Without osmolytes, *K*_d_ is ~13 μM. Addition of glycerol, sorbitol, and glucose, stabilizes the complex formation through decreasing *K*_d_ by 38%, 38%, and 16%, respectively (Fig. 4a). The shift of the equilibrium toward the complex demonstrates that the osmolytes stabilize the bound state more than the unbound state.

### Amide H/D exchange rate decrease by osmolytes

H/D exchange rates of amides have a long history of being used to characterize protein backbone h-bonds^44^. The rates of the slowest exchanged amides are determined by protein unfolding whereas the rates of the fastest exchanged amides are determined by direct interactions with solvent water^45^. For the amides with intermediate H/D exchange rate, the exchange is determined by the opening of protein local conformation^46^. To probe the osmolyte effect on the h-bonds of the PDZ3−CRIPT complex, H/D exchange rates for backbone amides were measured at 288 K by dissolving premixed PDZ3 (at the final concentration of 0.3 mM) and CRIPT peptide (1 mM) powder in a 50 mM sodium phosphate, pH 7.0, 100% D_2_O buffer. The 2D ^1^H−^15^N HSQC spectra were recorded consecutively. 45 fastest exchanged amides were not observed in the ^1^H−^15^N HSQC spectra whereas the 11 slowest exchanged h-bonded amides showed no H/D exchange in the 12 h experiments. The presence of 160 g/L glycerol, sorbitol, and glucose decreases the site-specific exchange rate of the remaining 21 residues with intermediate H/D exchanges, by an average of 34%, 36%, and 40%, respectively (Supplementary Table 3), suggesting that the local conformational opening is less frequent, consistent with the h-bond strengthening effect. Together with the PDZ3−peptide binding data, we can see that osmolytes shift the equilibrium towards the complex state likely through stabilizing the h-bonds.

## Discussion

**Fig. 5:**
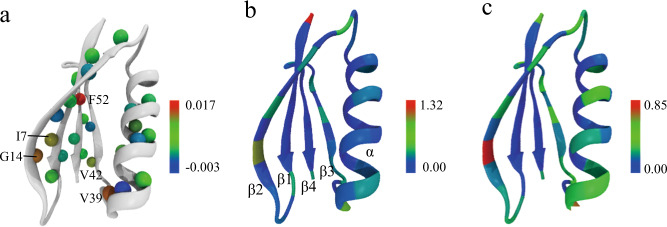
Site specific GB3 backbone h-bond perturbation by osmolytes and the backbone solvent accessibility. **a** The experimental Δ^3h^*J*_NC′_ averaged over glycerol, sorbitol, and glucose is mapped on the amide proton of the h-bond donor. Five h-bonds, I7→G14, G14→I7, V39→A34, V42→E56, and F52→K4 have Δ^3h^*J*_NC′_ larger than 0.01 Hz whereas G38→N35 is the only h-bond with Δ^3h^*J*_NC′_ < 0 Hz. Water (**b**), and glycerol (**c**) accessibility to the protein backbone is calculated by the number of water and glycerol molecules within 3.5 and 5 Å, respectively, from the backbone Cα of each residue (Supplementary Table 4, averaged over two 50 ns MD trajectories). The sorbitol and glucose accessibility is very similar to that of glycerol (Supplementary Fig. 2).

It is known that polyol and sugar osmolytes are preferentially excluded from the protein surface. Even so, the presence of osmolytes still perturbs protein intramolecular h-bonds. It has been found that TMAO can strengthen h-bonds of ubiquitin and reduce thermally induced h-bond weakening^47^, in line with observations in this work. Apparently, different protein h-bonds may respond differently to osmolytes. To elucidate such a difference, the experimental Δ^3h^*J*_NC′_ change by osmolytes is mapped on the structure of GB3 (Fig. 5). Since the Δ^3h^*J*_NC′_ values in glycerol, sorbitol, and glucose are correlated, the average of Δ^3h^*J*_NC′_ (over three osmolytes at 80 and 160 g/L) for each h-bond is reported. In all, 26 out of 27 h-bonds show a positive Δ^3h^*J*_NC′_ where a few h-bonds display larger Δ^3h^*J*_NC′_ (>0.01 Hz) than others. For example, I7 (donor)→G14 (acceptor) and G14→I7 are a pair of h-bonds between β1 and β2; V39→A34 is a h-bond between a short loop (39–41) and the end of the α helix; V42→E56 is an h-bond between β3 and β4 where E56 is the C-terminal residue; F52→K4 is a h-bond between β4 and β1 (Fig. 5). These are the hotspots with relatively large backbone h-bond perturbations. It is conceivable that the perturbation is caused by protein solvation change and/or interaction with osmolyte. The water and osmolyte accessibility calculated for each backbone Cα using MD trajectories shows a very similar pattern, which gives some hints about h-bond perturbation mechanism. The Cα of G14 has high solvent/osmolyte accessibility, consistent with shortening of the two h-bonds involving this residue. Similarly, V39, V42, and E56, which have larger h-bond perturbation, also show moderate solvent/osmolyte accessibility. However, residues such as T16, T18, and T44 have good solvent/osmolyte accessibility but rather small Δ^3h^*J*_NC′_. Apparently, solvent/osmolyte accessibility is not sufficient for a large h-bond perturbation. On the other hand, K4 in β1 and F52 in β4 have very low solvent/osmolyte accessibility, but the F52→K4 h-bond has a large Δ^3h^*J*_NC′_. It is not clear what causes this h-bond perturbation by osmolytes. It is likely that the perturbation is propagated from the D46→T51 h-bond which has high solvent/osmolyte accessibility. Unfortunately, this h-bond is too weak to measure experimentally. MD simulations suggest that the h-bond perturbation also changes the secondary structure percentage. The main change arises from the β-sheet, with the population increased from 40.3% (water) to 41.7% (glycerol), 41.4% (sorbitol), and 40.9% (glucose) whereas the α-helix population remains unchanged (Supplementary Fig. 3).

The shortening of protein h-bonds also suggests that the folded protein contracts in the presence of osmolytes. The average experimental Δ^3h^*J*_NC′_ of GB3 and TTHA over the three osmolytes is 0.004 Hz (80 g/L osmolytes) and 0.008 Hz (160 g/L osmolytes), corresponding to a shortening of *R*_NO_, the N–O distance of backbone N–H…O=C h-bond, by 0.0025 and 0.005 Å, respectively. Assuming the average *R*_NO_ is ~3 Å, addition of osmolytes only shortens this distance by 0.08% (80 g/L osmolytes) or 0.17% (160 g/L osmolytes). Contraction of disordered proteins^48–52^ and polymers^53,54^ by osmolytes has been well documented. Apparently, the same phenomenon occurs for folded proteins although the contraction is much smaller.

It is generally agreed that osmolytes stabilize protein folding mainly through the unfolded state destabilization by the solvation effect^8,16^. The protein h-bond shortening by osmolytes indicates an additional protein folding stabilization mechanism. Because the number of protein h-bonds in the folded state is far more than that in the unfolded state, the h-bond shortening helps stabilize the folded state more. The protein h-bond shortening would create an enthalpic contribution to protein stabilization, in addition to the solvation enthalpic gain of the denatured state, which has been reported by the protein stability measurements in different crowders^12,55^. Considering that the h-bond change is relatively small, the major osmolyte effect on protein stability may come from the denatured state destabilization. Recent studies have also shown that zwitterionic osmolytes can perturb electrostatic interactions^56–58^. It becomes clear that osmolytes are more than just inert crowders.

In conclusion, in this work, the polyol and sugar osmolyte effect on protein h-bonds is investigated. The three tested osmolytes, glycerol, sorbitol, and glucose, all are capable of shortening protein backbone h-bonds through the h-bond competition mechanism with solvent. Specifically, osmolytes weaken the protein solvent h-bonds to strengthen those within the protein. The h-bond shortening helps stabilize the PDZ3−CRIPT complex form where intermolecular h-bonds are critical for the binding. Our work provides direct evidences that polyol and sugar osmolytes can perturb protein h-bonds to affect protein function.

## Methods

### Sample preparation

The expression and purification protocols for GB3 have been described previously^59^. TTHA1718 was expressed in *E.coli* BL21 (DE3*) cells, transformed with a pET-11b vector containing the TTHA1718 gene, and purified using the procedure described in the literature^60^. PDZ3^303–395^ was prepared using the protocol described by Petit^41^. The C-terminal peptide from CRIPT (Ac-TKNYKQTSV-COOH) was synthesized by the APeptide Co. Ltd (Shanghai, China). Sorbitol, glycerol and glucose with purity >99.5% were purchased from Solarbio (Beijing, China).

### ^3h^*J*_NC′_ NMR measurement for GB3 and TTHA

All NMR experiments were carried out on a Bruker Avance 600 MHz spectrometer, equipped with a z-axis gradient, triple resonance, cryogenic probe. The through hydrogen bond J-coupling,^3h^

*J*_NC′_ was measured using the sequence developed by Cordier and Grzesiek^36^. In all, 3 mM ^2^H/^15^N/^13^C triple-labeled NMR sample (GB3 or TTHA) was prepared, containing 10 mM sodium phosphate (pH 6.5) and 0.3x protease cocktail inhibitor in 95%/5% H_2_O/D_2_O solution. The NMR experiments were performed with three osmolytes (glycerol, sorbitol, and glucose) at different concentrations (0, 80, and 160 g/L), respectively.

### Kd measurement for the PDZ3-CRIPT binding

The dissociation constant *K*_d_ was measured at 308 K, in the buffer of 20 mM sodium phosphate and 50 mM NaCl at pH 7.0. A series of 2D ^1^H-^15^N HSQC spectra were recorded for the ^15^N–labeled PDZ3^303–395^ (20 μM) by titrating aliquots of CRIPT with the final concentration of 0, 10, 20, 30, 40, 50, 60, 80, and 100 μM. The experiments were performed at the osmolyte (glycerol, sorbitol, and glucose) concentration of 0 and 160 g/L, respectively.

Chemical shift perturbation analysis was performed using a weighted vector combination of shifts, calculated by applying the formula Δ*ω* = [(∆*ω*_H_)^2^ + (0.1*∆*ω*_N_) ^2^]^0.5^ where ∆*ω*_H_ and ∆*ω*_N_ are the amide hydrogen and nitrogen chemical shift change caused by the addition of CRIPT peptide, and fitted to a single-site binding model by non-linear regression analysis to the following equation^61^.

$$\Delta {\upomega}_{{\mathrm{obs}}} = \Delta _{{\mathrm{max}}}\frac{{\left( {K_{\mathrm{d}} + \left[ {{L}} \right]_0 \, + \, \left[ {{P}} \right]_0} \right) - \sqrt {\left( {K_{\mathrm{d}} + \left[ {{L}} \right]_0 + \left[ {{P}} \right]_0} \right)^2-\left( {4\left[ {{P}} \right]_0\left[ {{L}} \right]_0} \right)} }}{{2[{{P}}]_0}}$$

where Δ*ω*_obs_ is the observed chemical shift, Δ_max_ is the maximum chemical shift change, [*L*]_0_ is the concentration of CRIPT peptide, [*P*]_0_ is the total protein concentration (20 μM).

### H-D exchange rate of PDZ3-CRIPT

Eight protein samples containing 300 μM PDZ3 and 1 mM CRIPT in 500 μL H_2_O were prepared and lyophilized. Sorbitol and glucose at the concentration of 160 g/L were dissolved into 99.9% D_2_O and lyophilized after 12 h for the H/D exchange usage. Then the sorbitol or glucose powder (with a final concentration of 160 g/L) was added into buffer consisting of 50 mM sodium phosphate at pH 7.0 in 99.9% D_2_O. Glycerol, at the same final concentration, was mixed with the D_2_O buffer directly. The addition of osmolytes can change the pH by ~0.2 pH unit, the pH was adjusted back to 7.0 by adding HCl or NaOH. The pH was measured using an InLab Micro Pro-ISM pH meter (Mettler Toledo). The lyophilized protein powder was then added into the 500 μL buffer (with or without osmolyte) for the H/D exchange experiment. 2D ^1^H-^15^N HSQC spectra were collected consecutively at 288 K. All the NMR spectra were processed and analyzed using NMRPipe and NMRDraw^62^. The exchange rates were obtained by fitting the 2D ^1^H-^15^N HSQC peak height of each residue to a two-parameter (for H/D exchanges in buffer, sorbitol, and glucose) or three-parameter (for H/D exchanges in glycerol) exponential function^63^.

### Molecular dynamics simulation

The starting coordinates of GB3 were downloaded from the RCSB Protein Data Bank (pdb: 2OED)^64^. The structures of osmolytes (glycerol, sorbitol, and glucose) were drawn and minimized in CHARMM-GUI^65^ (a web‐based graphical user interface for CHARMM, http://www. charmm‐gui.org). PACKMOL^66^ was used to add osmolytes randomly around GB3 which were then solvated in a cubic box filled with TIP3P water model. Two Na^+^ ions were added to neutralize the simulation systems, the final composition and size of the simulation boxes are listed in Supplementary Table 5.

MD simulations were carried out using the Gromacs 2019 program^67^, with the CHARMM36 force field^68^ for GB3 and the CHARMM general force field (CGenFF)^69^ for the osmolytes. The NPT ensemble was adopted in the initial relaxation of simulation systems with the pressure controlled at 1 bar by the Parrinello–Rahman method^70,71^, and the temperature maintained at 300 K by the Nose–Hoover thermostat^72^. The Particle-Mesh-Ewald Method^73,74^ was used to evaluate the contributions of the long-range electrostatic interactions. All bonds to hydrogen atoms in the protein were constrained by using the LINC algorithm^75^ whereas bonds and angles of water molecules were constrained by the SETTLE algorithm^76^. A time step of 2 fs was used.

Two ns of MD run was first performed by restraining the positions of heavy atoms of GB3 to their initial coordinates, followed by a 5-ns of MD only restraining the GB3 backbone. Then, the simulation system was equilibrated for 20 ns without any restraints. To improve the sampling efficiency, the REMD^43^ was used to produce the trajectory. The temperatures of replicas were set optimally using an online server (http://folding.bmc.uu.se/remd/)^77^, at 300.00, 303.78, 307.61, 311.46, 315.36, 319.29, 323.27, 327.27, 331.33, 335.43, 339.57, 343.75, 347.97, 352.23, 356.54, and 360.00 K, respectively. The ratios of exchange between neighboring replicas ranged between 19% and 31%. Each replica has a length of 50 ns. The trajectory was saved every 20 fs for data analyzes. The trajectories at 300 K were used for further analyses.

The through hydrogen bond *J*-coupling, ^3h^*J*_NC′_ (absolute value) was calculated using the equation^38^:

$$|J| = 59,000{\mathrm{exp}}( - 4R_{NO})$$

where *R*_NO_ is the distance between N and O, the heavy atoms of the h-bond donor and acceptor, respectively. The *R*_NO_ distance (Å) was obtained from the two 50 ns MD trajectories at 300 K. In the protein intra- and inter-molecular h-bond analysis, the cut-off for the donor-acceptor distance is 3.5 Å and the angle hydrogen–donor–acceptor is 30°. H-bonds were counted using the gmx hbond program in the Gromacs software.

### Statistics and reproducibility

All experiments were performed in duplicates for distinct samples except for the *K*_d_ measurement of the PDZ3-CRIPT binding which was performed using a series of ligand concentrations. The error of *K*_d_ was derived from the standard error of the mean of site-specific *K*_d_ of 12 residues (Supplementary Fig. 1). The simulations were performed twice with the same starting configuration but different initial velocities. The data are presented as mean ± SE.

### Reporting summary

Further information on research design is available in the Nature Research Reporting Summary linked to this article.

## Supplementary information

Supplementary Information

Reporting Summary

## Data Availability

All data generated or analyzed during this study are included in this published article (and its Supplementary Information files).
