# Supplementary material for: Polyol and sugar osmolytes can shorten protein hydrogen bonds to modulate function
Source: Commun Biol. 2020 Sep 23;3:528. doi: 10.1038/s42003-020-01260-1 (PMC7511342; doi:10.1038/s42003-020-01260-1)
Supplement: Supplementary file 1 — Supplementary Information [file 42003_2020_1260_MOESM1_ESM.pdf]

Polyol and Sugar Osmolytes can Shorten Protein Hydrogen Bonds to Modulate Function

Jingwen Li,[†,‡,⊥] Jingfei Chen,[†,‡,⊥] Liaoyuan An,[†,‡,§] Xiaoxiang Yuan,[†,‡,§] and Lishan Yao [*,†,‡]
[†] Key Laboratory of Biofuels and [‡] Shandong Provincial Key Laboratory of Synthetic Biology, Qingdao Institute of Bioenergy and Bioprocess Technology, Chinese Academy of Sciences, Qingdao, 266101, China
[§] University of Chinese Academy of Sciences, Beijing, 100049, China

Supporting Information

Supplementary Figures

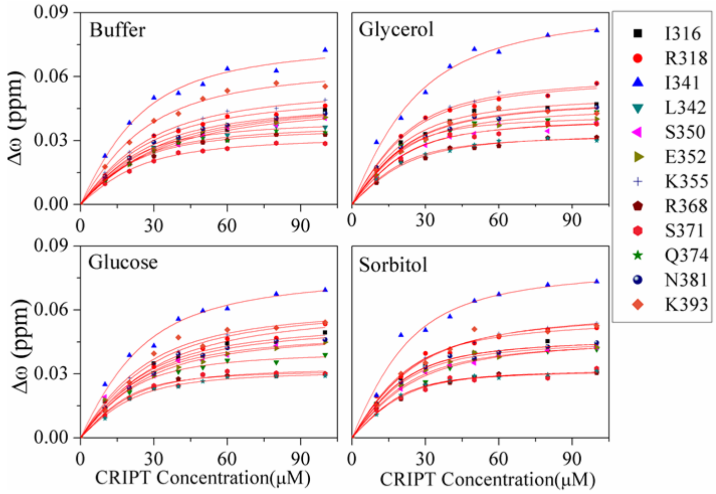

Supplementary Figure 1. Profiles of weighted chemical shift changes $\Delta\omega$ ($\Delta\omega = [(\Delta\omega_H)^2 + (0.1*\Delta\omega_N)^2]^{0.5}$) of different PDZ3 residues in the titration of CRIPT peptide. The red lines are the best fitted lines to eq. S1.

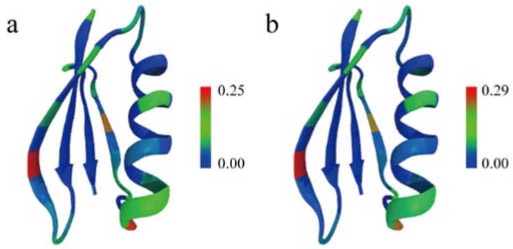

Supplementary Figure 2. Sorbitol (a) and glucose (b) accessibility to the protein backbone. The accessibility is calculated by the number of sorbitol and gluclose molecules within 5 Å from the backbone Cα of each residue and averaged over two 50 ns MD trajectories.

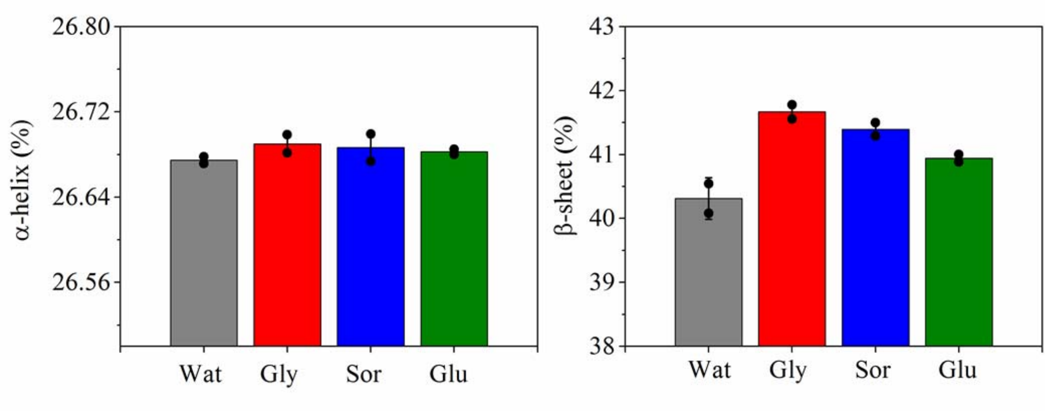

Supplementary Figure 3. Secondary structure percentage of GB3 in water and in the presence of different osmolytes. The percentages were calculated and averaged from 2 MD trajectories.

Supplementary Tables

Supplementary Table 1. Through hydrogen bond J-coupling, $^{3h}J_{NC'}$ measured for GB3 and TTHA at different osmolyte concentrations.[a]

GB3										
		Glycerol			Sorbitol			Glucose		
H^N	C′	0g/L	80g/L	160g/L	0g/L	80g/L	160g/L	0g/L	80g/L	160g/L
Y3	T18	0.647	0.648	0.650	0.647	0.655	0.659	0.652	0.656	0.660
K4	K50	0.399	0.404	0.411	0.398	0.402	0.405	0.400	0.405	0.410
L5	T16	0.681	0.684	0.688	0.681	0.688	0.691	0.689	0.692	0.694
I7	G14	0.554	0.564	0.579	0.557	0.563	0.573	0.561	0.562	0.574
N8	T44	0.605	0.617	0.633	0.604	0.612	0.617	0.611	0.586	0.591
G9	L12	0.367	0.375	0.375	0.374	0.384	0.384	0.368	0.379	0.372
G14	I7	0.230	0.240	0.255	0.232	0.236	0.243	0.231	0.242	0.250
T16	L5	0.404	0.405	0.405	0.406	0.409	0.403	0.410	0.408	0.413
T18	Y3	0.383	0.387	0.386	0.386	0.388	0.389	0.390	0.386	0.390
A20	M1	0.472	0.473	0.477	0.471	0.477	0.476	0.469	0.472	0.477
K28	E24	0.189	0.192	0.196	0.186	0.194	0.189	0.192	0.198	0.200
A29	T25	0.163	0.173	0.187	0.166	0.171	0.172	0.172	0.172	0.153
F30	A26	0.558	0.562	0.561	0.562	0.562	0.567	0.563	0.558	0.570
K31	E27	0.654	0.660	0.672	0.658	0.666	0.668	0.664	0.668	0.678
Q32	K28	0.207	0.212	0.220	0.205	0.206	0.207	0.208	0.215	0.213
Y33	A29	0.264	0.267	0.275	0.265	0.262	0.261	0.262	0.254	0.268
A34	F30	0.537	0.542	0.550	0.536	0.544	0.547	0.541	0.547	0.557
D36	Q32	0.561	0.567	0.573	0.562	0.570	0.567	0.565	0.572	0.575
N37	Y33	0.176	0.175	0.178	0.173	0.175	0.163	0.166	0.166	0.158
V39	A34	0.318	0.325	0.334	0.325	0.333	0.338	0.322	0.333	0.352
V42	T55	0.425	0.436	0.441	0.428	0.437	0.441	0.428	0.434	0.443

T44	T53	0.553	0.550	0.546	0.548	0.552	0.551	0.552	0.557	0.558
T51	D46	0.181	0.186	0.187	0.188	0.192	0.193	0.191	0.190	0.194
F52	K4	0.761	0.773	0.793	0.765	0.771	0.777	0.765	0.772	0.793
T53	T44	0.517	0.523	0.525	0.519	0.527	0.529	0.521	0.525	0.535
V54	V6	0.462	0.463	0.465	0.466	0.469	0.473	0.468	0.465	0.477
T55	V42	0.408	0.415	0.424	0.416	0.420	0.425	0.424	0.419	0.422
TTHA										
E7	K61	0.625	0.630	0.645	0.616	0.625	0.642	0.626	0.632	0.648
V18	S14	0.455	0.463	0.462	0.456	0.462	0.467	0.457	0.460	0.467
K20	M16	0.416	0.420	0.418	0.420	0.423	0.425	0.416	0.418	0.429
A21	A17	0.251	0.259	0.260	0.248	0.252	0.259	0.246	0.248	0.250
L22	V18	0.398	0.395	0.388	0.401	0.403	0.403	0.399	0.401	0.406
K23	T19	0.357	0.350	0.352	0.362	0.355	0.352	0.361	0.362	0.363
S34	E39	0.698	0.710	0.707	0.704	0.718	0.724	0.708	0.715	0.717
A40	L4	0.609	0.605	0.598	0.618	0.614	0.608	0.617	0.612	0.606
L41	E32	0.726	0.739	0.743	0.730	0.742	0.750	0.732	0.739	0.748
E43	K30	0.550	0.569	0.557	0.556	0.565	0.582	0.550	0.562	0.577
T45	G27	0.235	0.241	0.249	0.246	0.248	0.253	0.243	0.245	0.259
V52	P48	0.295	0.295	0.298	0.295	0.291	0.295	0.298	0.298	0.299
E56	V52	0.460	0.462	0.467	0.460	0.463	0.468	0.459	0.464	0.472
K61	E7	0.173	0.174	0.176	0.175	0.173	0.178	0.173	0.178	0.175
L65	K3	0.480	0.486	0.491	0.482	0.490	0.493	0.485	0.488	0.490

[a] ${}^{3h}J_{NC'}$ is the average from duplicate measurements. The measurement error of ${}^{3h}J_{NC'}$ is 0.004–0.006 Hz estimated from the standard deviation of ${}^{3h}J_{NC'}$ in the two measurements.

Supplementary Table 2. Statistics of hydrogen bonds in different simulation systems

Simulation systems	Number of h-bonds (GB3-water)	Number of h-bonds (GB3-osmolyte)	Total h-bonds	GB3-osmolyte h-bond percentage	${}^{3h}J_{NC}$
GB3	150.5±0.3	0	150.5±0.4	0	0.4748±0.0008
GB3/glycerol	105.5±0.1	35.9±0.1	141.4±0.1	25%	0.4889±0.0005
GB3/sorbitol	112.6±0.6	32.2±0.03	144.8±0.6	22%	0.4871±0.0009
GB3/glucose	113.8±0.5	31.0±0.01	144.8±0.5	21%	0.4831±0.0002

Supplementary Table 3. H/D exchange rates[a] for PDZ3 in the PDZ3-CRIPT complex.

Res.	Buffer	Glycerol	Sorbitol	Glucose
G303[1]	N/A	N/A	N/A	N/A
E304	f	f	f	f
E305	f	f	f	f
D306	f	f	f	f
I307	f	f	f	f
P308				
R309	f	f	f	f
E310	1.444±0.073	1.549±0.162	0.899±0.119	1.125±0.336
P311				
R312	1.057±0.045	0.545±0.197	0.596±0.006	0.537±0.066
R313	f	f	f	f
I314	0.007±0.001	0.003±0.001	0.007±0.001	0.003±0.001
V315	1.558±0.121	1.238±0.509	1.152±0.041	1.129±0.180
I316	s	s	s	s
H317	f	f	f	f
R318	f	f	f	f
G319	f	f	f	f
S320[1]	N/A	N/A	N/A	N/A
T321	f	f	f	f
G322	f	f	f	f
L323[1]	N/A	N/A	N/A	N/A
G324	f	f	f	f
F325	f	f	f	f
N326[2]	N/A	N/A	N/A	N/A
I327	f	f	f	f
I328	0.020±0.007	0.008±0.004	0.007±0.004	0.005±0.005
G329[1]	N/A	N/A	N/A	N/A
G330	f	f	f	f
E331	f	f	f	f
D332	f	f	f	f
G333	f	f	f	f
E334[1]	N/A	N/A	N/A	N/A
G335	f	f	f	f
I336	f	f	f	f
F337[2]	N/A	N/A	N/A	N/A
I338	f	f	f	f
S339[1]	N/A	N/A	N/A	N/A
F340	0.916±0.136	0.760±0.025	0.669±0.008	0.531±0.037
I341	f	f	f	f

L342	0.051±0.009	0.047±0.031	0.025±0.001	0.025±0.006
A343[1]	N/A	N/A	N/A	N/A
G344	f	f	f	f
G345	0.725±0.132	0.425±0.091	0.371±0.015	0.409±0.062
P346				
A347	1.094±0.169	0.680±0.110	0.588±0.062	0.538±0.038
D348	0.553±0.063	0.354±0.025	0.312±0.019	0.337±0.078
L349	0.976±0.158	0.664±0.022	0.576±0.047	0.534±0.136
S350	f	f	f	f
G351	f	f	f	f
E352	f	f	f	f
L353[1]	N/A	N/A	N/A	N/A
R354	f	f	f	f
K355	f	f	f	f
G356[2]	N/A	N/A	N/A	N/A
D357	s	s	s	s
Q358	0.007±0.001	0.005±0.001	0.006±0.001	0.006±0.002
I359	s	s	s	s
L360	s	s	s	s
S361	s	s	s	s
V362	s	s	s	s
N363	f	f	f	f
G364	f	f	f	f
V365	0.006±0.001	0.004±0.000	0.005±0.001	0.005±0.001
D366	f	f	f	f
L367	0.026±0.004	0.024±0.000	0.020±0.001	0.020±0.002
R368	1.451±0.112	1.422±0.056	1.056±0.130	1.677±0.250
N369	f	f	f	f
A370	f	f	f	f
S371	f	f	f	f
H372	f	f	f	f
E373	f	f	f	f
Q374	f	f	f	f
A375	0.008±0.003	0.005±0.004	0.003±0.001	0.002±0.002
A376	0.0105±0.002	0.005±0.002	0.004±0.001	0.005±0.000
I377	0.0105±0.004	0.005±0.003	0.004±0.001	0.006±0.003
A378	s	s	s	s
L379	0.004±0.002	0.002±0.002	0.004±0.001	0.002±0.001
K380	0.010±0.001	0.004±0.004	0.004±0.001	0.004±0.003
N381	f	f	f	f
A382	f	f	f	f
G383	f	f	f	f
Q384	f	f	f	f

T385	f	f	f	f
V386	0.080±0.003	0.059±0.004	0.053±0.001	0.055±0.001
T387[2]	N/A	N/A	N/A	N/A
I388	s	s	s	s
I389	s	s	s	s
A390	s	s	s	s
Q391	s	s	s	s
Y392	f	f	f	f
K393	f	f	f	f
P394				
E395	f	f	f	f

[a.] The error is estimated from duplicate measurements.

[1.] The exchange rates are not available due to overlap.

[2.] The residue has no signal on hsqc spectrum.

f. The exchange rates are too fast to be measured.

s. The exchange rates are too slow to be measured.

Supplementary Table 4. Number of water and glycerol molecules around backbone Cα of GB3 from MD simulations.

Res	Water	Glycerol
1	1.319072	0.542383
2	0.062975	0.097361
3	0.052379	0.04938
4	0.004398	0.02439
5	0	0
6	0.0004	0.0002
7	0.001	0
8	0.109156	0.054778
9	0.412435	0.068373
10	0.152139	0.247901
11	0.236705	0.288884
12	0.195122	0.238505
13	0.089764	0.178329
14	0.965214	0.851459
15	0.010196	0.138545
16	0.408237	0.30028
17	0.006597	0.061176
18	0.348061	0.316273
19	0.038784	0.106757
20	0.531387	0.402439
21	0.12575	0.27409
22	0.210916	0.213515

23	0.012595	0.097561
24	0.264694	0.32467
25	0.181327	0.113754
26	0.005398	0.0002
27	0.0012	0.0004
28	0.101359	0.136146
29	0.262495	0.416833
30	0.0012	0.003399
31	0.05018	0.105558
32	0.089964	0.148141
33	0.17513	0.2499
34	0.0012	0.004398
35	0.218113	0.270692
36	0.332867	0.37585
37	0.198721	0.334466
38	0.873651	0.704318
39	0.09916	0.076569
40	0.371052	0.435226
41	0.29928	0.290084
42	0.213115	0.197321
43	0.021192	0.180128
44	0.382247	0.5002
45	0.045982	0.179328
46	0.348461	0.378848
47	0.015994	0.14994
48	0.305278	0.473611
49	0.180928	0.262495
50	0.0012	0.011196
51	0.001799	0.0006
52	0.012995	0
53	0.0004	0.001999
54	0.039184	0
55	0.008796	0.004798
56	0.383047	0.269892

Supplementary Table 5. Number of molecules in MD simulation systems.

Simulation systems	Number of osmolytes	Number of waters	Box size (nm)	Mass weight of osmolytes (g/L)
GB3	0	3373	4.76×4.76×4.76	0
GB3/glycerol	361	1898	4.77×4.82×4.77	503
GB3/sorbitol	184	1976	4.73×4.73×4.73	526
GB3/glucose	185	2043	4.74×4.70×4.71	527
